# Accuracy of intraocular lens calculations in eyes with keratoconus

**DOI:** 10.1097/j.jcrs.0000000000001088

**Published:** 2022-10-28

**Authors:** Magali M.S. Vandevenne, Valentijn S.C. Webers, Maartje H.M. Segers, Tos T.J.M. Berendschot, David Zadok, Mor M. Dickman, Rudy M.M.A. Nuijts, Adi Abulafia

**Affiliations:** From the University Eye Clinic, Maastricht University Medical Center+, Maastricht, the Netherlands (Vandevenne, Webers, Segers, Berendschot, Dickman, Nuijts); Department of Ophthalmology, Shaare Zedek Medical Center, Jerusalem, Israel (Zadok, Abulafia).

## Abstract

To compare the prediction accuracy of the Barrett True-K for keratoconus with standard formulas (SRK/T, Barrett Universal II, and Kane) and the Kane keratoconus formula.

**Setting:**

Shaare Zedek Medical Center, Jerusalem, Israel, and University Eye Clinic, Maastricht, the Netherlands.

**Design:**

Multicenter retrospective case series.

**Methods:**

Eyes with stable keratoconus undergoing cataract surgery were included. The predicted refractions were calculated for SRK/T, Barrett Universal II, Barrett True-K for keratoconus (predicted and measured), Kane, and Kane adjusted for keratoconus formulas. Primary outcomes were prediction error (PE), absolute error (AE), and percentage of eyes with PE ±0.25 diopters (D), ±0.50 D, and ±1.00 D. Subgroup analyses were performed based on the severity of the keratoconus.

**Results:**

57 eyes were included in the study. The PE was not significantly different from zero for SRK/T, Barrett True-K (predicted and measured), and Kane keratoconus formulas (range 0.09 to 0.22 D, *P* > .05). The AE of Barrett True-K predicted (median 0.14 D) and Barrett True-K measured (median 0.10 D) were significantly lower from Barrett Universal II (median 0.47 D) and Kane (median 0.50 D), *P* < .001.

**Conclusions:**

The Barrett True-K formulas for keratoconus had higher prediction accuracy as compared with new generation formulas and a similar prediction accuracy as compared with the Kane keratoconus formula.

## Abstract 2

Recently, 2 keratoconus-adjusted formulas, Kane keratoconus and Barrett True-K for keratoconus, were introduced to calculate IOL power in patients with keratoconus. These formulas improve the prediction accuracy in patients with keratoconus.

Intraocular lens (IOL) prediction accuracy in patients with keratoconus is challenging.^1–10^ This is because the assumed fixed ratio of anterior to posterior corneal curvature is not maintained in eyes with keratoconus, leading to errors in the estimated total corneal power (TCP) and the predicted effective lens position (ELP), resulting in hyperopic prediction error with most formulas that increases as keratoconus worsens. Moreover, the multifocal cornea in keratoconus makes it difficult to obtain consistent corneal power measurements and subjective refraction.

Savini et al. reported that the SRK/T formula has better accuracy compared with other standard formulas.^4^ Recently, 2 keratoconus-adjusted formulas were introduced. The Kane keratoconus formula was found to be more accurate compared with standard formulas in a large series.^6^ The Barrett True-K for keratoconus, using either predicted or measured posterior corneal power (PK), was evaluated in a small cohort of toric IOLs in select patients with regular central corneas.^10^ The aim of this study was to independently compare the prediction accuracy of the Barrett True-K for keratoconus with standard formulas (SRK/T, Barrett Universal II, and Kane) and the Kane keratoconus formula.

## METHODS

A retrospective chart review of consecutive subjects with keratoconus who had undergone cataract surgery between August 2012 and April 2021 was performed at 2 separate centers: (1) Shaare Zedek Medical Center, Jerusalem, Israel, and (2) University Eye Clinic Maastricht, Maastricht, the Netherlands. Inclusion criteria were stable keratoconus, postoperative corrected distance visual acuity (CDVA) of at least 20/40, uneventful cataract surgery with an IOL positioned in the capsular bag, no other corneal diseases, and no previous ocular surgery or trauma. If both eyes of a patient were eligible for inclusion, 1 eye was randomly selected.^11,12^ The study adhered to the tenets of the Declaration of Helsinki.

Preoperative evaluation included CDVA, manifest refraction, corneal tomography (Pentacam, Oculus Optikgeräte GmbH, software v. 1.9r532, Carl Zeiss Meditec AG), and biometry using either partial coherence interferometry-based or swept-source optical coherence tomography-based devices (IOLMaster 500, software v. 7.3-7.7, and IOLMaster 700, software v. 1.70-1.80, respectively, Carl Zeiss Meditec AG). Uncorrected distance visual acuity (UDVA), CDVA, and manifest refraction were obtained at least 1 month postoperatively. Patients who used contact lenses were instructed to stop wearing their soft, scleral, and rigid gas-permeable contact lenses at least 1 week, 2 weeks, and 4 weeks before their preoperative biometry measurements, respectively. All cataract surgeries were performed under topical anesthesia by experienced surgeons using a standard phacoemulsification technique through a 2.2 mm or 2.4 mm clear corneal incision by either a superior or a temporal approach.

### IOL Calculation and Comparison

The IOL power was calculated using the following formulas: SRK/T, Barrett Universal II, Barrett True-K for keratoconus (using either predicted or measured PK), Kane, and Kane adjusted for keratoconus. For all IOL power calculations, standard anterior-based K-values taken by the biometry measurements were used (n = 13 375), whereas PK and central corneal thickness values were obtained from the corneal tomography measurements. The SRK/T prediction values were obtained from the biometry printouts. The predicted postoperative spherical equivalent (SE) for Barrett Universal II and Barrett True-K was calculated using the Asia-Pacific Association of Cataract and Refractive Surgeons website.^13,14^ The predicted postoperative SE for the Kane and Kane adjusted for keratoconus formulas was calculated using the online Kane calculator.^15^

When available, the IOL constants derived from the IOLCon website (https://iolcon.org/) were used to perform IOL calculations. IOL constants not included on the IOLCon website were provided by the IOL manufacturers. The prediction error (PE) was defined as the difference between the measured and predicted postoperative SE. A negative PE indicates a more myopic outcome compared with the predicted refraction, whereas a positive PE indicates a more hyperopic result. The mean prediction error (MPE), mean absolute prediction error (MAE), median absolute prediction error (MedAE), and percentages of eyes with PE within 0.25 diopters (D), 0.50 D, and 1.00 D were calculated for each formula.

### Keratoconus Classification

Subgroup analysis was performed based on the severity of keratoconus using a slightly modified version of the Amsler-Krumeich classification: Patients were classified as stage I when the mean standard keratometry (IOL Master) was ≤48 D, stage II when the mean keratometry was <53 D, and stage III when the mean keratometry was ≥53 D.^16^

### Statistical Analysis

Data were collected in Excel 2016 (Microsoft Corp.) and transferred to SPSS (v. 25.0, IBM Corp.) for data analysis. Descriptive statistics were presented as the mean and standard deviation (SD). The *Journal of Cataract and Refractive Surgery* statistical analysis guidelines for comparing different methods for IOL calculations were followed.^11^ The Kolmogorov-Smirnov test was used to check for normality. In case of nonnormal distribution, nonparametric tests were used. One sample *t* test or Wilcoxon signed rank test was used to analyze if the MPE were significantly different from zero. Absolute errors of the different formulas were assessed with the Friedman test; for pairwise comparisons, we used the Wilcoxon test. The Cochran *Q* test was used to compare the percentage of eyes within certain PE between the formulas, and for pairwise comparisons, we used the McNemar test. The Holm-Bonferroni correction method was applied for multiple comparisons.

## RESULTS

A total of 87 eyes of 57 patients were eligible for inclusion. Thirty eyes were excluded after randomly selecting 1 eye in bilateral cases. The mean age at surgery was 64 years, and 58% were female. Table 1 presents the demographics of the study cohort. A higher-than-normal mean axial length (24.9 mm) and anterior chamber depth (3.47 mm) were found. According to the modified Amsler-Krumeich classification, 36 eyes (63%) were categorized as stage I, 17 eyes (30%) as stage II, and 4 eyes (7%) as stage III. Supplementary Table 1 (available at http://links.lww.com/JRS/A739) presents the number of eyes implanted with each IOL design and the constants used.

**Table 1.** Demographics of study cohort

Parameter	All stages	Stage I	Stage II	Stage III	*P* value
n	57	36	17	4	
Age (y)	64.8 ± 9.1	63.8 ± 9.5	67.9 ± 8.1	60.8 ± 8.8	.206
M/F	24/33	19/17	4/13	1/3	.102
Right/left eye	25/32	19/17	4/13	2/2	.130
Kmean (D)	46.6 ± 3.6	44.4 ± 1.7	49.4 ± 1.2	54.9 ± 1.6	<.001*
AL (mm)	24.9 ± 1.7	24.9 ± 1.6	25.2 ± 2.2	24.1 ± 0.9	.528
ACD (mm)	3.47 ± 0.37	3.40 ± 0.33	3.59 ± 0.40	3.56 ± 0.48	.183
Preop CDVA (logMAR)	0.44 ± 0.31	0.36 ± 0.29	0.52 ± 0.25	0.77 ± 0.47	.013
Postop CDVA (logMAR)	0.12 ± 0.13	0.06 ± 0.08	0.18 ± 0.11	0.35 ± 0.26	<.001*

ACD = anterior chamber depth; AL = axial length; K = keratometry

* Significant after Holm-Bonferroni correction

**Figure 1.**
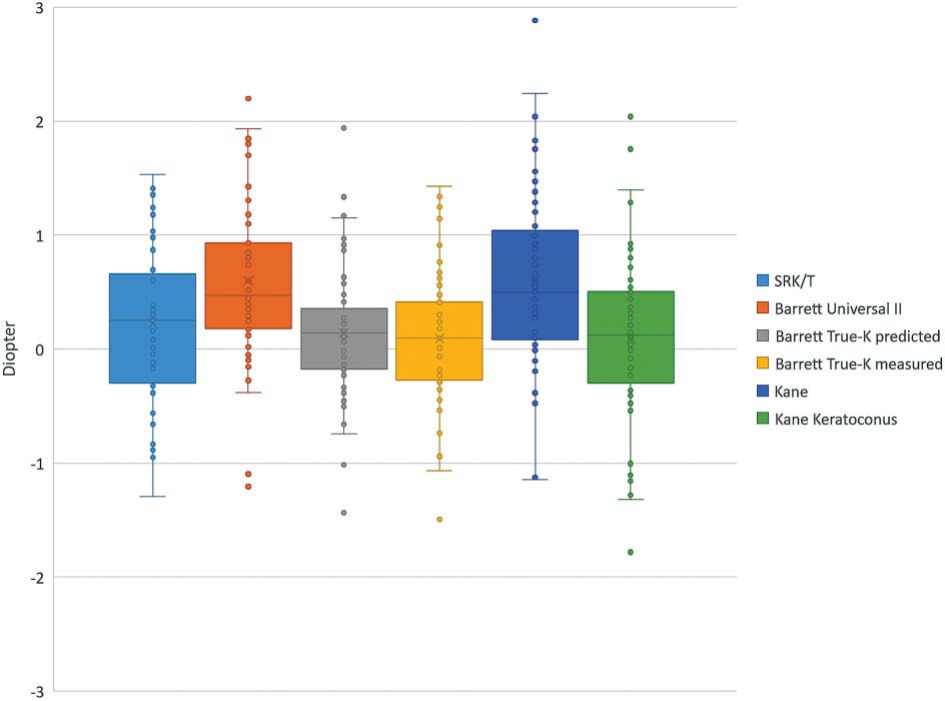
Distribution of prediction error.

Table 2 presents the MPE, MAE, and MedAE, and respective SDs for all patients. Figure 1 shows the distribution of the PE for each formula. The MPE was significantly different from zero for the Barrett Universal II and the Kane formula (0.60 D, adjusted *P* < .001). The MPE was not significantly different from zero for SRK/T, Barrett True-K (predicted and measured), and Kane keratoconus formulas. The AEs of the Barrett True-K predicted (MedAE 0.14 D) and Barrett True-K measured (MedAE 0.10 D) were significantly different from Barrett Universal II (MedAE 0.47 D) and Kane (MedAE 0.50 D, adjusted *P* < .001, Supplementary Table 2, available at http://links.lww.com/JRS/A740). No other statistically significant differences between formulas were found (adjusted *P* ≥ .01).

**Table 2.** Overview of prediction error outcomes in different IOL calculation formulas

Formula	MPE ± SD	MAE ± SD	MedAE
SRK/T	0.22 ± 0.66	0.56 ± 0.42	0.25
Barrett Universal II	0.60 ± 0.71*	0.72 ± 0.58	0.47
Barrett True-K predicted	0.14 ± 0.58	0.43 ± 0.42	0.14
Barrett True-K measured	0.09 ± 0.58	0.44 ± 0.38	0.10
Kane	0.60 ± 0.77*	0.74 ± 0.63	0.50
Kane keratoconus	0.09 ± 0.74	0.55 ± 0.50	0.13

MAE = mean absolute prediction error; MedAE = median absolute prediction error; PE = mean prediction error

All values expressed in diopters

* One-sample *t* test, significant after Holm-Bonferroni correction

**Figure 2.**
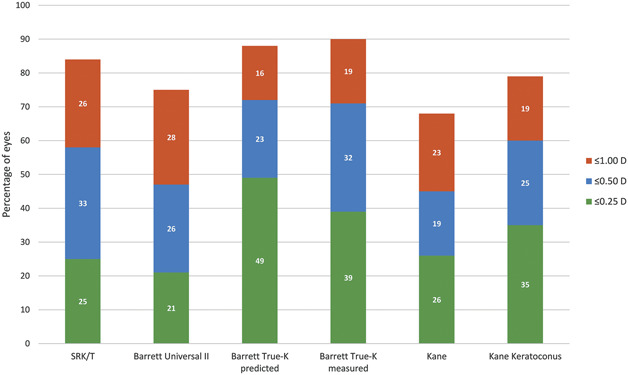
Percentage of eyes within prediction error.

Figure 2 shows the percentages of eyes with PE within ±0.25 D, ±0.50 D, and ±1.00 D for each formula. The Barrett True K predicted had the highest percentage of eyes within 0.25 D and 0.50 D (49% and 72%, respectively). For the ±0.25 D category, this was significantly higher compared with SRK/T and Barrett Universal II (adjusted *P* = .007 and .006, respectively). For the ±0.5 D category, this was significantly higher compared with Kane and Barrett Universal II (adjusted *P* = .003). The Barrett True-K measured had the highest percentage of eyes within 1.0 D (90%). This was significantly higher compared with Kane (adjusted *P* = .002, Supplementary Tables 3.1 to 3.3, available at http://links.lww.com/JRS/A741, http://links.lww.com/JRS/A742, http://links.lww.com/JRS/A743).

### Subgroup Analysis

Table 3 presents the MPE, MAE, and MedAE for each keratoconus stage. The MPE was significantly different from zero using the Barrett Universal II, Kane, and Kane keratoconus formulas for stage I (adjusted *P* ≤ .01), and for the Barrett Universal II and Kane formula for stage II (adjusted *P* < .001) and stage III (adjusted *P* = .005 and .009, respectively). The MedAE was lowest using either Barrett True-K predicted or Barrett True-K measured formula for all stages. No statistically significant differences in MedAE were found between formulas for all stages, except when comparing the Barrett True-K predicted or Barrett True-K measured formulas with the Barrett Universal II and Kane formulas in stage II keratoconic eyes (adjusted *P*-value < .01) (see Supplementary Table 4, available at http://links.lww.com/JRS/A744). In general, an increase in MPE, MAE, and MedAE was seen with advancing stages of the disease with the exception of the Kane keratoconus formula that had more myopic MPE as the stage of keratoconus advanced.

**Table 3.** Overview of prediction error outcomes in different IOL calculation formulas per stage

Formula	Stage I			Stage II			Stage III		
	MPE ± SD	MAE ± SD	MedAE	MPE ± SD	MAE ± SD	MedAE	MPE ± SD	MAE ± SD	MedAE
SRK/T	0.22 ± 0.54	0.47 ± 0.34	0.34	0.11 ± 0.70	0.56 ± 0.41	0.39	0.72 ± 1.34	1.36 ± 0.12	1.33
Barrett Universal II	0.35 ± 0.65*	0.54 ± 0.50	0.40	0.91 ± 0.55*	0.92 ± 0.54	0.86	1.57 ± 0.43*	1.57 ± 0.43	1.76
Barrett True-K predicted	0.05 ± 0.59	0.39 ± 0.44	0.22	0.28 ± 0.58	0.49 ± 0.40	0.39	0.46 ± 0.46	0.54 ± 0.29	0.56
Barrett True-K measured	0.01 ± 0.58	0.44 ± 0.37	0.29	0.19 ± 0.59	0.43 ± 0.43	0.32	0.49 ± 0.19	0.49 ± 0.19	0.44
Kane	0.30 ± 0.66*	0.52 ± 0.50	0.37	0.89 ± 0.48*	0.90 ± 0.45	0.92	2.04 ± 0.66*	2.04 ± 0.66	1.90
Kane keratoconus	0.30 ± 0.66*	0.52 ± 0.50	0.37	−0.15 ± 0.74	0.58 ± 0.47	0.51	−0.75 ± 0.65	0.75 ± 0.65	0.84

MAE = mean absolute prediction error; MedAE = median absolute prediction error; PE = mean prediction error

All values expressed in diopters

* One-sample *t* test, significant after Holm-Bonferroni correction

## DISCUSSION

Recently, 2 new keratoconus-specific IOL formulas were introduced to improve IOL power prediction accuracy in keratoconus eyes: the Kane keratoconus and Barrett True-K keratoconus formulas.^6,17^ The Kane keratoconus formula aims to provide more appropriate corneal power measurements and to reduce the influence of corneal power on ELP. It uses a modified corneal power derived from anterior corneal radii of curvature in steep corneas to compensate for the altered anterior to posterior radii ratio of curvature in keratoconus eyes.^6^ The Barrett True-K incorporates the posterior corneal power (predicted or measured) and central corneal thickness to estimate the TCP in keratoconus.^18^

The number of studies investigating keratoconus-adjusted IOL formulas is limited.^6,10^ In our study, we retrospectively compared the prediction accuracy of the Barrett True-K keratoconus with 3 conventional formulas (SRK/T, Barrett Universal II, and Kane) and the Kane keratoconus formulas in eyes with stable keratoconus. Our results suggest a higher prediction accuracy of the Barrett True-K formulas as compared with new generation formulas, and similar to the Kane keratoconus formula.

It is well known that standard formulas (except for SRK/T) tend to yield hyperopic results in eyes with keratoconus due to overestimation of the corneal power. Melles at al. described that SRK/T leads to significant myopic PE in normal eyes as the average keratometry increases.^19^ The tendency of the SRK/T toward myopic PE in higher corneal powers counterbalances the hyperopic tendency seen in patients with keratoconus as suggested by Kane et al.^6^ Our results confirm previous studies reporting the SRK/T yields better results in keratoconus eyes.^4^ However, advanced formulas such as Barrett Universal II and Kane tend to yield hyperopic PE in keratoconus.^1,2,4,10^ Designated formulas try to overcome this challenge by estimating the TCP either using a prediction algorithm (Kane keratoconus and Barrett True-K predicted) or using direct measurements of the posterior corneal curvature (Barrett True-K measured).^6,10^ Indeed, we found that the PEs did not differ significantly from zero for these designated formulas. Nevertheless, we found prediction becomes less accurate with more advanced keratoconus (Table 3). One novelty of our study is the head-to-head comparison of these designated formulas in a representative cohort of keratoconus eyes.

Although the Barrett True-K using measured posterior corneal curvature has a theoretical advantage over a mathematical model (Barrett True-K predicted), our results suggest that the use of direct measurements of the posterior cornea did not improve the prediction accuracy of the Barrett True-K formula. This finding contrasts with Ton et al. and may be explained by their inclusion criteria of eyes with regular central cornea and agreement across different devices.^10^ One possible explanation for our findings is lower accuracy of posterior corneal curvature measurements in eyes with keratoconus. Further studies are needed to optimize such measurements in these eyes.

Our study has several limitations. Sample size for the subgroup analyses was small, especially for stage III keratoconus. Second, optimization of the IOL formula constants was not performed for this unique cohort.^6,11,12^ Moreover, due to the retrospective nature of our study which included patients over a large period, 2 different optical biometers have been used over the years. Each biometer has a different way of measuring the anterior keratometry and over different zones, which might be important especially in eyes with keratoconus because the keratometry varies significantly over the different zones of the cornea. Further studies are needed to evaluate these potential differences in predicted accuracy between biometry devices in this population. Another limitation of our study is that it included data collected by multiple surgeons in 2 different Ophthalmology departments. Finally, although the follow-up period was at least 30 days (average 84 days), refraction can fluctuate up to 6 months, especially in thin corneas with keratoconus.^20^

In conclusion, the Barrett-True K formulas were more accurate as compared with the Barrett Universal II and the Kane new generation IOL formulas and had a slight advantage over the SRK/T formula (Barrett-True K predicted) and comparable with the Kane keratoconus formula. Keratoconus-specific IOL formulas should be the preferred choice in patients with keratoconus. Further studies including larger cohorts and more patients with advanced keratoconus are needed to better assess prediction accuracy in these challenging eyes.

**WHAT WAS KNOWN**

- IOL power calculation in eyes with keratoconus (KCN) is challenging.
- The Kane KCN and Barrett true-K (predicted and measured) formulas are designed to deliver a higher prediction accuracy in eyes with KCN.

**WHAT THIS PAPER ADDS**

- The Barrett True-K using the predicted or measured posterior keratometry was more accurate compared with new generation formulas.
- In our cohort, the use of direct or predicted measurements of the posterior cornea did not improve the prediction accuracy of the Barrett True-K formula.
